# Harmonization of Brain Diffusion MRI: Concepts and Methods

**DOI:** 10.3389/fnins.2020.00396

**Published:** 2020-05-06

**Authors:** Maíra Siqueira Pinto, Roberto Paolella, Thibo Billiet, Pieter Van Dyck, Pieter-Jan Guns, Ben Jeurissen, Annemie Ribbens, Arnold J. den Dekker, Jan Sijbers

**Affiliations:** ^1^Department of Radiology, Antwerp University Hospital, University of Antwerp, Antwerp, Belgium; ^2^imec–Vision Lab, University of Antwerp, Antwerp, Belgium; ^3^Icometrix, Leuven, Belgium; ^4^Physiopharmacology, University of Antwerp, Antwerp, Belgium

**Keywords:** harmonization, normalization, diffusion MRI, multi-site, inter-scanner, review

## Abstract

MRI diffusion data suffers from significant inter- and intra-site variability, which hinders multi-site and/or longitudinal diffusion studies. This variability may arise from a range of factors, such as hardware, reconstruction algorithms and acquisition settings. To allow a reliable comparison and joint analysis of diffusion data across sites and over time, there is a clear need for robust data harmonization methods. This review article provides a comprehensive overview of diffusion data harmonization concepts and methods, and their limitations. Overall, the methods for the harmonization of multi-site diffusion images can be categorized in two main groups: diffusion parametric map harmonization (DPMH) and diffusion weighted image harmonization (DWIH). Whereas DPMH harmonizes the diffusion parametric maps (e.g., FA, MD, and MK), DWIH harmonizes the diffusion-weighted images. Defining a gold standard harmonization technique for dMRI data is still an ongoing challenge. Nevertheless, in this paper we provide two classification tools, namely a feature table and a flowchart, which aim to guide the readers in selecting an appropriate harmonization method for their study.

## Introduction

Diffusion-weighted magnetic resonance imaging (dMRI) is an MRI technique in which the image contrast is related to the diffusion of water molecules inside tissues. dMRI has brought great innovation to neuroimaging analysis, since it enables non-invasive probing of brain microstructure. Nevertheless, many studies using diffusion data rely on small sample sizes, leading to poor reproducibility of results. Fortunately, research is evolving toward large multicenter studies with the aim of increasing statistical power. However, the success of a joint analysis is highly dependent on the comparability of the multi-site data.

Diffusion data of the same subject obtained at different sites and/or acquired at different time points can be different due to local and/or temporal scanner characteristics resulting in a high inter- and intra-scanner variability (Vollmar et al., 2010; Grech-Sollars et al., 2015; Nencka et al., 2017). These variabilities may arise from a range of factors, such as hardware (scanner manufacturer, field strength, transmitter/receiver coils, magnetic field inhomogeneities, etc.), reconstruction algorithms (SENSE, GRAPPA, etc.), acquisition parameters (voxel size, number of gradient directions, echo time, repetition time, etc.), and image quality [signal to noise ratio (SNR), etc.] (Alexander et al., 2006; Ni et al., 2006; Jones, 2010; Vollmar et al., 2010). All these factors affect the final diffusion signal intensity and consequently the diffusion metrics, preventing reliable multi-site and/or longitudinal diffusion studies (Pfefferbaum et al., 2003; Vollmar et al., 2010; Mirzaalian et al., 2018).

In literature, many conflicting inferences have been reported between studies, in which findings based on small distinct cohorts are used to generalize conclusions for an entire population, without considering intra- and inter-site differences (Button et al., 2013; Kelly et al., 2018; Smith and Nichols, 2018). To determine the site effects on diffusion data, a number of studies examined diffusion phantom data to detect scanner related variabilities (Teipel et al., 2011; Zhu et al., 2011; Walker et al., 2013; Pullens et al., 2017; Timmermans et al., 2019). Up to 7% of inter-site variability in diffusion metrics was demonstrated in phantoms (Teipel et al., 2011; Palacios et al., 2017). However, using parameters obtained from phantom data to correct human data is not advised due to the structural complexity of human biological tissue (Karayumak et al., 2019).

Previous research has established that inter-site variability is non-uniform across the white matter of the human brain, with a variability up to 5% in diffusion metrics of major brain tracts (Vollmar et al., 2010; Grech-Sollars et al., 2015; Nencka et al., 2017). Recently, investigators have examined the reproducibility of multi-shell diffusion images in a multi-site study involving traveling subjects (Tong et al., 2019). A 7.7% median inter-center coefficient of variation was estimated for the track density maps in whole white matter among the subjects. These inter-site variabilities in diffusion metrics are similar to the changes due to pathologies. For example, in the work of Kumar et al. (2009), it was shown that the variability in diffusion metrics in the corpus callosum between controls, mild Traumatic Brain Injury (TBI) and moderate TBI patients, are of the same order as intra-scanner changes. Furthermore, a quantitative study by Mahoney et al. reported longitudinal changes in diffusion metrics in dementia patients compared to controls in the same order of the site variabilities (Mahoney et al., 2015). From these findings, we can infer that it is crucial to reduce the variability across multi-center diffusion data.

Inter-site variability can be reduced by acquiring data with scanners from the same manufacturer at each site and using similar acquisition parameters (Vollmar et al., 2010; Fox et al., 2012; Cannon et al., 2014). However, diffusion parameters of subjects scanned using the same acquisition protocol may still differ significantly across sites (Nyholm et al., 2013; Jovicich et al., 2014; Mirzaalian et al., 2015). These differences may come from several sources, such as sensitivity of head coils, imaging gradient non-linearities, magnetic field inhomogeneities and other scanner related factors. Hence, there is a substantial need for robust harmonization techniques (Jenkins et al., 2016; Jovicich et al., 2019). The overall concept of harmonization methods is to apply statistical or mathematical concepts to reduce unwanted site variability while maintaining the biological content. In the last decade a multitude of harmonization methods have been developed.

For this review, we have categorized the brain dMRI methods in two main groups depending on the data-format used as input for harmonization. The first category uses calculated diffusion (para)metric maps, such as Fractional Anisotropy (FA), Mean, Axial and Radial Diffusivity (MD, AD, and RD, respectively), Kurtosis Anisotropy (KA), Mean, Axial, and Radial Kurtosis (MK, AK, and RK, respectively), as input (i.e., diffusion parametric map harmonization; DPMH). While the second category uses diffusion weighted images (DWI) as input (i.e., diffusion weighted image harmonization; DWIH).

To the authors’ knowledge, no previous study has provided an extensive report of diffusion harmonization methods. In this review paper, a comprehensive overview of those methods is presented, including an investigative analysis of their strengths and weaknesses. DPMH and DWIH methods reported since 2009 are described. This paper is organized as follows. Section “Literature Search” describes the search mechanism used for selecting the literature on brain diffusion data harmonization. In Section “Requirements for Harmonization,” the requirements for harmonization are specified. Sections “Diffusion Parametric Map Harmonization Methods” and “Diffusion Weighted Image Harmonization Methods” depict the DPMH and DWIH harmonization methods reported in the literature. Section “Discussion” then presents an overview of the main characteristics of the methods and a guideline that helps the user to select an adequate harmonization method for her/his data. Finally, in Section “Conclusion” conclusions are drawn.

## Literature Search

Two authors (MSP and RP) independently performed a literature search across two databases (PubMed and Google Scholar) using combinations of the following search terms: “harmonization,” “harmonisation,” “normalization,” “normalisation,” “multi-site,” “multi-center,” “inter-site,” “intra-scanner,” “diffusion,” “MRI,” “DTI,” “meta-analysis,” “covariates,” “spherical harmonics,” “deep learning.” Besides the usual search engines, additional important papers were selected by checking the reference lists of identified relevant publications on data harmonization. After removing the duplicates, all identified articles were screened by title and abstract. Studies were included if they described diffusion harmonization methods and concepts.

## Requirements for Harmonization

For the majority of dMRI harmonization procedures, co-registration is of crucial importance. Co-registration of diffusion images aims to find spatial transformations to map different images to a common reference space, allowing direct comparison of various image properties. Prior to harmonization, a voxel-by-voxel correspondence between multiple diffusion volumes is needed, in order to minimize errors in subsequent calculations. In particular, voxel-wise DPMH and DWIH approaches require all subjects to be in the same space in order to extract common features that are site-related rather than anatomically specific. The common space can be a study-specific template or a standard brain atlas template, as for example, the ICBM152 template of the Montreal Neurological Institute (MNI) space^1^. Many tools are available for registering diffusion images, such as Advanced Normalization Tools (ANTs; Avants et al., 2011), FMRIB Software Library (FSL; Jenkinson et al., 2012), and elastix (Klein et al., 2010; Shamonin et al., 2014).

Additionally, a dataset with a balanced number of subjects per site is advised for robust harmonization. Many DPMH and DWIH methods use these subjects to efficiently learn a set of so-called mapping parameters used to characterize the differences between the images across scanners. Additionally, an important requirement, especially for DWIH methods, is the availability of training data, i.e., matched subjects across sites for obtaining the mapping parameters between sites. Age, gender, handedness, and socio-economic status need to be matched among the subjects to remove the statistical differences at group level. Moreover, for some machine learning techniques, there is a need for DWI data of individual subjects that are scanned at different sites, within a small interval of time, to train a network to recognize site-related underlying inter-scanner/inter-site differences in the characteristics of the images to harmonize.

Overall, for all the methods, it is highly recommended to use a balanced dataset and to co-register the diffusion images or maps to a common template. The recommendations are to assure that statistical differences are only due to hardware, software and protocol differences, and ensure spatial compatibility intra- and inter-subjects during the harmonization procedure. Furthermore, each method has its own specifications and limitations that are described in the following sections.

## Diffusion Parametric Map Harmonization Methods

Diffusion parametric map harmonization methods perform particular transformations on the diffusion parametric maps that enable data pooling and reduction of unwanted intra- and inter-site variability. For a joint analysis of multi-site diffusion metric maps that have been estimated using a given diffusion model [e.g., diffusion tensor imaging (DTI), diffusion kurtosis imaging (DKI), neurite orientation dispersion and density imaging (NODDI), etc.], statistical or mathematical DPMH methods can be applied. The purpose of these methods is to perform joint statistical analysis on multi-site data. It can be performed in two ways: (1) without modifying the original diffusion parametric maps (see Subsection “Modeling Inter-Site Variability Within the Statistical Analysis”); (2) by modifying the parametric maps with *a posteriori* analysis (see Subsection “Harmonizing the Parametric Maps Based on Regression of Covariates”). DPMH methods allow to pool DWI parametric maps obtained from different diffusion acquisition schemes (diffusion directions, *b*-values, repetition time, echo time, etc.). The DPMH methods described below are meta-analysis, mega-analysis, and regression of covariates.

### Modeling Inter-Site Variability Within the Statistical Analysis

#### Meta-Analysis

Meta-analysis is a popular statistical analysis technique in biomedical research that combines results of independent multi-site and/or longitudinal studies. The general concept is to perform a group-wise statistical analysis separately for each site, followed by a weighted combination of effect size over the different studies to strengthen conclusions about the research question (Zhu et al., 2019). Meta-analysis is useful to pool retrospective data with sample sizes that are too small to draw valid conclusions independently (Petitti, 1994).

**FIGURE 1:**
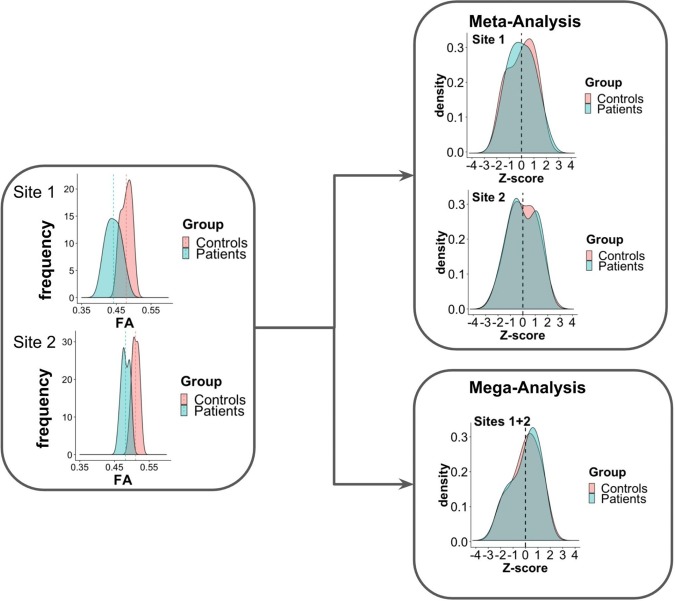
Scheme of meta- and mega-analysis. FA measures from sites 1 and 2, for two groups of subjects: controls and patients. The FA frequency for each group is estimated for each of the sites. Meta-analysis performs the statistical evaluation between groups for each site separately, followed by a weighted combination of its statistical results, while in mega-analysis a weighted statistical evaluation is performed for all sites jointly.

Figure 1 presents an example of meta-analysis in which statistical inferences are obtained independently per site from the FA maps of different groups of subjects. As a first step, an intra-site statistical analysis is performed. The resulting statistical scores (e.g., *z*-score) of the metric of interest (e.g., FA) can then be weighted by each site’s sample size or with respect to an estimate of precision, such as effect size (Salimi-Khorshidi et al., 2009), to obtain the final statistical score. In contrast to this approach, the overall statistical score can also be obtained by modeling site as a random effect (Worsley, 2002; Beckmann et al., 2003; Woolrich et al., 2004). For example, in the work of Teipel et al. (2012), meta-analysis was used to investigate FA and MD differences between dementia patients and controls in a multi-site study, taking scanner effects into account. Voxel-based *t*-statistics were converted to *z*-scores after which a variance component analysis was applied, effectively reducing effects of site (random effect), age and gender (fixed effects).

One of the main advantages of meta-analysis is the possibility to pool data from small/underpowered studies to derive robust conclusions. It is also the only way to pool studies for which only aggregated data are reported (e.g., group difference statistics or the mean FA per region of interest) and for which the whole brain images are not available. However, one drawback is that if the statistics performed in the individual studies are biased by study size, the population estimate will be also affected. Another disadvantage is that the statistical analysis should first be performed separately for each diffusion metric of interest.

#### Mega-Analysis

In contrast to meta-analysis, mega-analysis refers to a technique of summarizing the statistics from the individual subjects of all sites to jointly evaluate population group differences (Jahanshad et al., 2013; Zhu et al., 2019). As depicted in Figure 1, in mega-analysis group-difference statistics are not calculated for each site separately. Instead, group differences are identified by a site-weighted combination of the statistical scores from all individuals jointly.

When the individual diffusion data (e.g., FA) is available per subject, the measures can be pooled to calculate the effect size across the entire group in a mega-analysis. To take into account the variability due to site differences, the site effect can be modeled using a mixed linear model statistical approach (or another statistical method to analyze the dataset), just as in meta-analysis.

While not directly harmonizing the imaging data itself, mega-analysis allows a joint analysis of two (or more) datasets to evaluate a common characteristic in the population (Jahanshad et al., 2013; Kochunov et al., 2014; Zhu et al., 2019). Some limitations in this approach are that the size of the cohort may not be sufficient to capture the variance of the entire population, pre-processing steps could be very different for each site (if the FA maps are computed independently), and the statistical analysis has to be performed separately for each variable (e.g., FA, MD, AD, and RD).

Meta-and mega-analysis have successfully been adopted in the field of neuroimaging by the Enhancing NeuroImaging Genetics through Meta-Analysis (ENIGMA) consortium (Jahanshad et al., 2013; Kochunov et al., 2014). The general concept of the harmonization method proposed by the ENIGMA-DTI group is that each site preprocesses the diffusion metric maps (e.g., FA) separately. The statistical scores are harmonized using meta- or mega-analysis, to improve data comparability and robustness. Findings of the ENIGMA-DTI group indicate that results obtained by meta- and mega-analysis may differ, in favor of the latter. In multi-center studies with a moderate amount of variation between cohorts, a mega-analysis statistical framework appears to be the better approach to investigate structural neuroimaging data, showing greater stability and higher power for jointly analyzing the data (Kochunov et al., 2014). Nonetheless, when the individual diffusion metric maps are not available, meta-analysis could serve as a valuable alternative. However, meta-analysis should be performed carefully and one should take into account cohort trends (Kochunov et al., 2014).

### Harmonizing the Parametric Maps Based on Regression of Covariates

**FIGURE 2:**
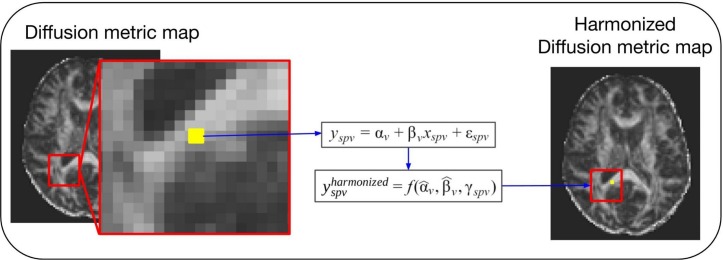
General scheme of a voxel-wise regression of covariates harmonization approach. For these methods the voxel intensity of the diffusion metric maps (*y*_spv_, the intensity for a specific site *s*, subject *p* and voxel *v*) is modeled as a combination of a voxel-wise intercept (α_v_), a voxel-wise slope (β_v_) multiplied by a model-specific dependent variable (*x*_spv_), and an error component (ε_spv_). Each of the regression of covariates approaches will have a different model and dependent variable to describe the biological and site-related effects on the diffusion metric intensities. Next, the estimated coefficients are used to compute the new harmonized diffusion intensity values ($y_{\text{spv}}^{\text{harmonized}}$).

Covariates, also known as explanatory variables, are variables that may affect the estimate of the diffusion metric under study. These covariates can be variables of clinical interest or unwanted confounding variables, such as MR hardware (e.g., scanner manufacturer, field strength, and coils), software, acquisition parameters (e.g., echo time, repetition time, *b*-value, and gradient directions) or image quality. One way to handle unwanted variability due to confounding factors is the use of regression models (Pourhoseingholi et al., 2012). This approach is illustrated in Figure 2. After fitting a regression model to the diffusion values, adjusted values can be derived that no longer contain the effect of the covariates. The use of the regression of covariates harmonization approach to correct for variability in software and hardware has been reported extensively in the literature (Forsyth et al., 2014; Venkatraman et al., 2015; Fortin et al., 2016, 2017; Pohl et al., 2016; Timmermans et al., 2019). Regression of covariates methods can be divided into two categories: global harmonization methods and voxel-wise harmonization methods. Both classes are described below.

The methods present different options for harmonizing diffusion metric maps (e.g., FA and MD maps). For briefness, we use the notation $y_{\text{spv}}^{\text{method}}$ to denote the diffusion metric measure *y* harmonized by a specific *method*, at site *s*, for subject *p* and voxel *v*.

#### Global Harmonization

##### Human-phantom based harmonization (HuP)

A straightforward approach for data harmonization is to apply scanner-specific correction factors derived from human phantom data (i.e., a group of individuals scanned at multiple scanners/sites within a short period of time) (Pohl et al., 2016). One scanner type is defined as the reference (*R*) and the other as the target (*T*). The goal is to correct the diffusion metric maps of the target site. For this purpose, a correction factor (*F*) is calculated as the ratio of the mean value (across the human phantoms) of the diffusion metric in the reference and target, respectively: $F=\frac{\sum_{p}{\bar{y}}_{\text{p}}^{\text{R}}/N}{\sum_{p}{\bar{y}}_{\text{p}}^{\text{T}}/N}$, where ${\bar{y}}_{\text{p}}^{\text{R}}$ and ${\bar{y}}_{\text{p}}^{\text{T}}$ are the mean metric value across the white matter voxels for human phantom *p* at the reference and target site, respectively, and *N* is the number of human phantoms. Successively, once the scanner-specific correction factors are determined, metric maps *y* for subject *p* and voxel *v* scanned in the target scanner (*y*_spv_) are scaled by the appropriate correction factor in order to obtain the HuP-harmonized diffusion metric maps: $y_{\text{spv}}^{\text{HuP}}=y_{\text{spv}}F$.

The main advantages of the correction factor are its simple derivation and the fact that it has been demonstrated to correct for differences that are likely attributable to the MR system manufacturer (Pohl et al., 2016). However, human phantom datasets from multiple sites are required. Moreover, a unique correction factor per scanner type only partially reduces the harmonization problem due to its intrinsic non-linearity, i.e., scanner type differences are not uniform but vary in a highly non-linear fashion across the brain (Karayumak et al., 2019).

##### Hardware-phantom based harmonization (HaP)

Timmermans et al. (2019) presented global multi-site harmonization models, using phantom data acquired at multiple centers in a longitudinal study. For this study, dedicated diffusion single-strand phantoms were developed by HQ Imaging (Heidelberg, Germany). The study aimed to build a comprehensive model for the variability of FA. Protocol-specific and site-specific effects were included in the models, considering hardware (scanner vendor and head coil), software, acquisition parameters (bandwidth, TE, and TR), image quality (signal-to-noise ratio and mean residual), as fixed predictor variables, and site as random predictor variable, taking into account that fixed predictors relate to effects that are constant across all individuals, and random predictors relate to effects that vary across individuals.

Different models were proposed to describe the diffusion metric values *y*_p_ of the phantoms *p* considering the differences between acquisitions and were evaluated via the combination of the fixed and random predictors (*x*_p_ and *z*_p_, respectively): $y_{\text{p}}^{\text{HaP}}=\beta_{0}+\beta_{\text{n}}x_{\text{p}}+b_{0p}+b_{\text{np}}z_{\text{p}}+\varepsilon_{\text{p}}$, where β_0_ is the fixed intercept, β_n_ the fixed effects slope, *b*_0p_ the random intercept per phantom, *b*_np_ the random slope per phantom, and ε_p_ the error. In order to find the most comprehensive model for the diffusion metric data, many linear mixed effects models were evaluated by the Akaike information criterion (AIC). The selection of model parameters was based on three model categories: protocol-specific intercept, protocol-specific intercept with quality effects, and protocol-specific intercept with protocol-specific quality effects. Each model was further divided into submodels depending on the included variables. AIC is used to select which model best describes the variations in the metric intensities. The results showed that scanner manufacturer, SNR, head coil, bandwidth and TE are the covariates that best describe the sources of variability in the inter-site phantom data, and should be used to harmonize the diffusion metric maps of multi-center studies.

The use of hardware phantoms for harmonization has several advantages. Hardware phantoms can be scanned multiple times, for a longer time, and their images do not suffer from motion artifacts. The phantom content is controllable and remains stable over time. Duplicated phantoms can be easily obtained by several sites, obviating transport. The main drawback of hardware phantom based harmonization is that such phantoms do not fully represent the complexity of the human brain, and therefore have different, intra- and inter-scanner variabilities. Obviously, voxel-wise harmonization (cf., Section “Voxel-Wise Harmonization”) of brain dMRI is not possible using phantom data.

##### Global scaling (GS)

In the global scaling method presented by Fortin et al., 2017, a linear model is used to correct the site effect on the diffusion metric maps (Fortin et al., 2017). The estimated location (θ_s,location_) and scale (θ_s,scale_) model parameters, per site *s*, encapsule the variabilities in the diffusion metric maps due to site effects. They are estimated by fitting a linear regression model: ${\bar{Y}}_{\text{s}}=\theta_{s,\mathrm{location}}+\theta_{s,\mathrm{scale}}\bar{Y}+\varepsilon_{\text{s}}$, where ${\bar{Y}}_{\text{s}}$ is an *n*_v_ ×1 vector containing the average diffusion metric intensity per voxel for the number of voxels *n*_v_ computed over all subjects of site *s*, $\bar{Y}$is an *n*_v_ x1 vector containing the average diffusion metric intensity per voxel for the number of voxels *n*_v_ computed over all subjects of all sites together (considered a reference), and ε_s_ is the residual error. From the estimated parameters, the harmonized diffusion metric maps are calculated as: $y_{\text{spv}}^{\text{GS}}=\frac{y_{\text{spv}}-{\hat{\theta }}_{s,\mathrm{location}}}{{\hat{\theta }}_{s,\mathrm{scale}}}$.

The main advantage of global scaling is that it takes into account information from all sites. Some disadvantages are that the removal of site effects can also remove biological variability, and that it does not account for spatial heterogeneity of the site effects in the brain.

#### Voxel-Wise Harmonization

##### Removal of Artificial Voxel Effect by Linear regression (RAVEL)

The Removal of Artificial Voxel Effect by Linear regression (RAVEL) method (Fortin et al., 2016) uses voxels in the cerebrospinal fluid (CSF) voxels as control region. The CSF-voxels are used for harmonization because their diffusion metric intensities are unassociated with disease or other clinical factors and are theoretically only influenced by site-related variabilities. In this method, the voxel-wise intensity of the diffusion metric maps (*y*_spv_) is described as a combination of four components: the average intensity in the sample (α1^t^), the known clinical covariates of interest (β*X*^t^), the unknown site-related factors (γ*Z*^t^) and a residual (*R*): *y*_spv_ = α1^t^ + β*X*^t^ + γ*Z*^t^ + *R*. Where the symbol *t* indicates the transpose operation, *y*_spv_ is the *v* × *p* matrix containing the registered and normalized voxel intensities for *v* voxels and *p* subjects, α1^t^ is a *v* × 1 vector containing the average voxel intensity per site, *X* is a *p* × *k* matrix containing for each subject *p* the correspondent biological covariates *k*, β is the coefficient matrix associated with *X*, *Z* is a *p* × *m* matrix containing for each subject *p* the associated *m* unwanted coefficient factors and γ is the coefficient matrix associated with *Z*.

The CSF voxels are used to estimate the unknown/unwanted factors (*Z*^t^) by assuming that α and β are null for the CSF since there is no association between control voxels and clinical features. Thus, the CSF diffusion intensities ($y_{\text{spv}}^{\text{CSF}}$) are described as: $y_{\text{spv}}^{\text{CSF}}=\gamma_{\text{CSF}}Z^{\text{t}}+R_{\text{CSF}}$. Singular value decomposition is used to obtain the first latent factors (*w*_1sp_) from the CSF voxels, representing the site-related variability common to all voxels. Next, the voxel-wise RAVEL coefficients (ψ_v_) are estimated fitting the linear regression model to the voxel-wise diffusion intensities (*y*_spv_) and the first latent factors (*w*_1sp_): *y*_spv_ = α_v_ + ψ_v_*w*_1sp_ + ε_spv_, where ε_spv_ is the residual error. Lastly, the RAVEL-harmonized diffusion metric map intensities are computed: $y_{\text{spv}}^{\text{RAVEL}}=y_{\text{spv}}-{\hat{\psi }}_{\text{v}}w_{1sp}$.

An advantage of the RAVEL method is that it is a voxel-wise harmonization method that uses intra-subject information that is not affected by disease (CSF control region) for improving comparability between subjects. However, if these control regions do not carry the information about the inter-site variability and/or are related to the parameter of interest, then the correction may remove relevant biological information, becoming a disadvantage to use this method in such cases.

##### Surrogate Variable Analysis (SVA)

Surrogate Variable Analysis (SVA) identifies and estimates unknown, unmodeled or unwanted sources of variation from the data (Leek et al., 2012; Fortin et al., 2017). The so-called batch effects can be defined as measurements of unwanted variability that have qualitatively different behavior across conditions and are unrelated to the biological or scientific variables in a study (Leek et al., 2010). In the context of multi-site harmonization, SVA is particularly useful when it is not known which datasets belong to which site. Through singular value decomposition, the data is decomposed into a set of *m* surrogate variables (*z*_1_,…,*z*_m_). Variables with the largest variance, and which are not covarying with *a priori* defined factors of interest such as age, gender or diagnosis, are then regressed out of the data. The voxel-wise SVA coefficients (Φ_mv_) are estimated by fitting the surrogate variables (*z*_msv_, for surrogate variable *m*, site *s* and voxel *v*) and the original diffusion metric intensities (*y*_spv_, for site *s*, subject *p* and voxel *v*) to the linear regression model: $y_{\text{spv}}=\alpha_{\text{v}}+\sum_{n=1}^{m}\Phi_{\text{nv}}z_{\text{nsp}}+\varepsilon_{\text{spv}}$, where α_*v*_ is the voxel-wise overall measure of the diffusion metric and ε_spv_ is the residual error. Next, the SVA-harmonized diffusion metric map intensities ($y_{\text{spv}}^{\text{SVA}}$) are computed as: $y_{\text{spv}}^{\text{SVA}}=y_{\text{spv}}-\sum_{n=1}^{m}{\hat{\Phi }}_{\text{nv}}z_{\text{nsp}}$.

Surrogate variable analysis is implemented in the SVA package for R, and is applicable voxel-wise (Leek et al., 2012). A strong point is that it estimates all common sources of latent variation, without needing to know their exact origin (e.g., site). Nonetheless, if this inherent variation is related to biological variability (e.g., patients in site A, controls in site B) then SVA is not appropriate.

##### Combined association test (ComBat)

The combined association test (ComBat) uses regression of covariates for data harmonization (Fortin et al., 2017). It started as a batch effect correction tool (similar to SVA) used in genomics, in which the batch effect is known (Johnson et al., 2007). It is a powerful and fast alternative for SVA in cases where site is an *a priori* known factor.

ComBat describes the non-harmonized diffusion metric in each voxel (*y*_spv_, for site *s*, subjects *p* and voxel *v*) by an adjustment model that consists of the following terms: an overall measure of the diffusion metric (α_v_), the product of a design matrix (*X*_sp_) containing the covariates of interest (e.g., gender and age) and the vector of corresponding regression coefficients (β_v_), a term representing the so-called additive site effects (γ_sv_) and, finally, the product of a normally distributed error term (ε_spv_) and a factor representing the so-called multiplicative site effects (δ_sv_): *y*_spv_ = α_v_ + *X*_sp_β_v_ + γ_sv_ + δ_sv_ε_spv_. The site-specific parameters of the adjustment model are assumed to have parametric prior distributions, being a normal distribution for the additive factor (γ_sv_) and an inverse gamma distribution for the multiplicative factor (δ_sv_). The parametric distributions are estimated from the data, using an empirical Bayes framework to decrease the variance of the site effects. It assumes that all voxels share a common distribution, and are used to infer the properties of the site-effects. Subsequently, ComBat-harmonized diffusion parameter maps are created based on the estimated additive and multiplicative factors ($\gamma_{\text{sv}}^{{}^{*}}$ and $\delta_{\text{sv}}^{{}^{*}}$, respectively): $y_{\text{spv}}^{\text{ComBat}}=\frac{y_{\text{spv}}-{\hat{\alpha }}_{\text{v}}-X_{\text{sp}}{\hat{\beta }}_{\text{v}}-\gamma_{\text{sv}}^{*}}{\delta_{\text{sv}}^{*}}+{\hat{\alpha }}_{\text{v}}+X_{\text{sp}}{\hat{\beta }}_{\text{v}}$.

It was reported that the ComBat harmonization method preserves between-subject biological information (Fortin et al., 2017). However, a limitation of this method is that the optimization procedure assumes the site effect parameters to follow a particular parametric prior distribution (Gaussian and Inverse-gamma), which might not generalize to all scenarios or measures. Moreover, it is not clear how non-linearities in the signal due to site effects propagate through the preprocessing techniques, as well as model fitting procedures.

## Diffusion Weighted Image Harmonization Methods

Diffusion parametric map harmonization methods for data pooling and joint analysis, meta- and mega-analysis and regression of covariates, have been reported extensively in the literature. Nonetheless, the harmonization of diffusion metric maps has several drawbacks, as described in section 4 for each of the methods. Recall that one of the main drawbacks is the lack of knowledge on how the scanner-specific non-linearities propagate in the diffusion model fit, possibly affecting the harmonization procedure of the diffusion metric maps. Recently, the use of the dMRI intensity signal has been proposed to perform model-free harmonization approaches. These methods are categorized as DWIH (Mirzaalian et al., 2015; Koppers et al., 2018; Huynh et al., 2019; Karayumak et al., 2019; Tax et al., 2019). DWIH methods rely on mapping the DWI images to a reference space. An overview of these DWIH approaches is given below. The methods described are the rotation invariant spherical harmonics method, machine learning algorithms, and the method of moments.

### Rotation Invariant Spherical Harmonics (RISH)

The use of rotation invariant spherical harmonics (RISH) for dMRI signal harmonization has been first proposed by Mirzaalian et al. (2015) and several improvements to this method have been presented since then (Mirzaalian et al., 2016, 2018; Karayumak et al., 2019).

The core idea of the RISH method is to map the diffusion weighted imaging (DWI) data from a target (*T*) site to a reference (*R*) site. The voxel-wise DWI signal intensity *S* = [*s*_1_, …, *s*_g_]^t^, along *g* unique directions, can be compactly represented in a spherical harmonics (SH) basis: $S\approx \sum_{i}\sum_{j}C_{\text{ij}}Y_{\text{ij}}$, composed by SH basis functions (*Y*_ij_) and their corresponding coefficients (*C*_ij_) of order *i* and degree *j*, with *j* = 1,2,…,2i + 1. The RISH features, per harmonic order, are extracted from the estimated SH coefficients as: RISHi=||Ci|2|=∑j=12i+1(Cij2).

**FIGURE 3:**
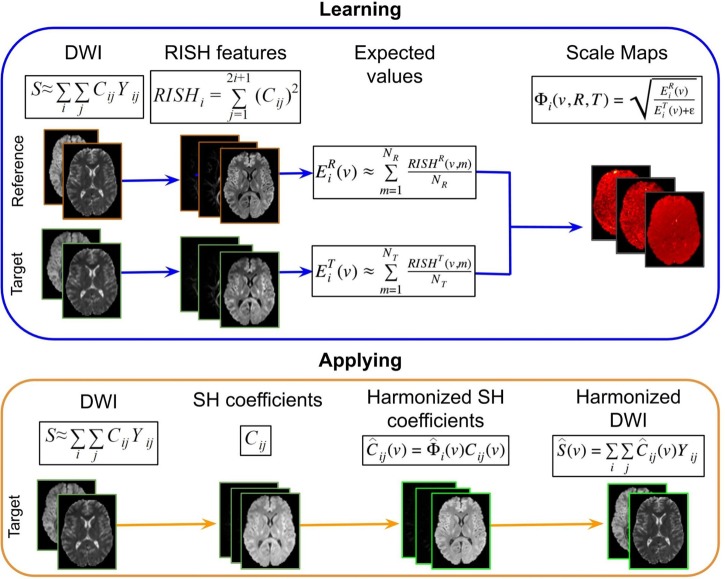
Representation of the RISH harmonization approach. Consider the purpose of modifying the DWI acquired in a target site, to correspond to the DWI acquired in the reference site. In the learning part using matched subjects, the RISH features are computed in native space from the DWI for the two data sets separately: reference (*R*) and target (*T*) sites. Then RISH features are transformed to a common space, the expected values are calculated per site *s* and per harmonic order *i* ($E_{\text{i}}^{\text{s}}$), after which the scale maps are calculated (Φ_i_). The scale maps, which are computed for each harmonic order *i*, represent the transformation of the RISH features from target to reference site. Next, in the application step, the SH coefficients from the target site are calculated, the scale maps are warped into native space and applied to the SH coefficients, creating harmonized SH coefficients in native space. Those are transformed back to the signal intensity domain, obtaining the harmonized DWI. Thus, harmonized DWI from the target site can be jointly analyzed with the ones from the reference site.

The harmonization procedure, which is illustrated in Figure 3, consists of two parts: (1) learning scale maps between sites from training data and (2) applying the learned scale maps to harmonize all DWI of the target site. The learning part is performed using training data that is a subset of subjects that are matched by age and gender for both sites. From the DWI, the RISH features are calculated and used to create a multivariate template, per *b*-value shell. In template space, the voxel-wise expected value per site *s* and per harmonic order *i* [$E_{\text{i}}^{\text{s}}\left(v\right)$] of RISH features is calculated as the sample mean over the number of training subjects (*N*_s_): $E_{\text{i}}^{\text{s}}\left(v\right)\approx \sum_{p=1}^{N_{s}}RISH_{\text{i}}^{\text{s}}\left(v,p\right)/N_{\text{s}}$, where *s* represents the site, *v* the voxel location in template space and *p* the training subject. Then, voxel-wise scale maps (Φ_i_) are computed for each harmonic order i: $\Phi_{\text{i}}\left(v,R,T\right)=\sqrt{\frac{E_{\text{i}}^{\text{R}}\left(v\right)}{E_{\text{i}}^{\text{T}}\left(v\right)+\varepsilon }}$. Next, in the application part, the scale maps are used to calculate the harmonized SH coefficients of the target data per harmonic order: ${\hat{C}}_{\text{ij}}\left(v\right)=\hat{{\text{Φ}}_{\text{i}}}\left(v\right)C_{\text{ij}}\left(v\right)$. Next, the image is transformed from SH domain back to the intensity signal domain [$\hat{S}\left(v\right)$] using the harmonized SH coefficients: $\hat{S}\left(v\right)=\sum_{i}\sum_{j}{\hat{C}}_{\text{ij}}\left(v\right)Y_{\text{ij}}$.

Rotation invariant spherical harmonics has many advantages, the most important one being that it harmonizes the raw dMRI signal in a model-independent manner. The mapping captures only site-related differences, preserving the between-subject biological variation and fiber orientation (Karayumak et al., 2019). However, a limitation is that it requires dMRI data with similar acquisition parameters across sites. It also requires the same number of matched controls that are scanned in both reference and target sites to obtain the scale maps.

### Machine Learning

In the past decade, several diffusion data harmonization methods have been developed employing a machine learning approach, such as sparse dictionary learning (SDL) and deep learning (DL).

#### Sparse Dictionary Learning (SDL)

Sparse dictionary learning is a representation learning method aiming at representing the input data as a linear combination of elements (the sparse dictionary), thus reducing the complexity of the harmonization problem (Mairal et al., 2010). The dictionary elements are small patches of spatial and angular image features (e.g., 3 × 3 × 3 × 5 voxels) that are learnt from the data itself. From a large set of random features, SDL extracts the common features with which full images can be reconstructed. The idea behind applying SDL for harmonization is that when a sparse dictionary can be constructed from data originating from multiple sites, the learnt imaging features will not include features of inter-site variability, as those are not common across the input data. Reconstructing dMRI data with a sparse dictionary, would then effectively harmonize the data (St-Jean et al., 2016; St-Jean et al., 2017).

An advantage of this method is that modeling a signal with such a sparse decomposition (sparse coding) is very effective in detecting salient regions that are related to the more informative areas. However, a disadvantage is that, depending on the interest points and the type/resolution of the image, sometimes only a few regions are detected.

#### Deep Learning (DL)

**FIGURE 4:**
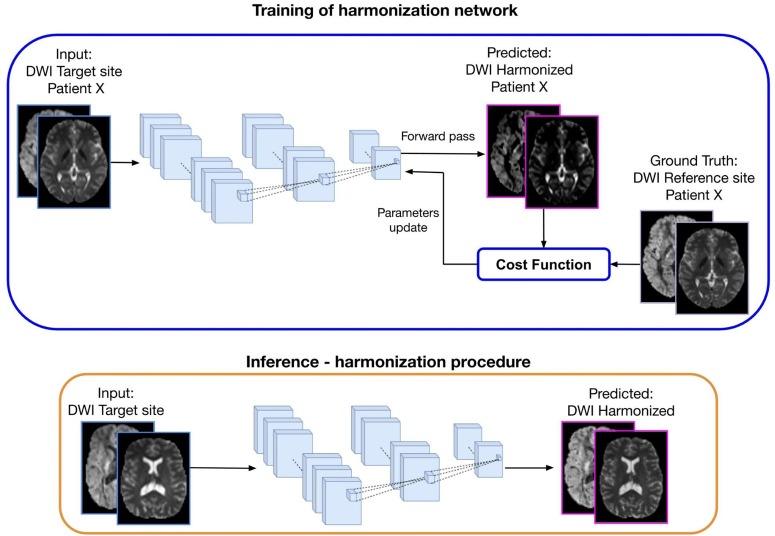
Representation of a deep learning approach for diffusion data harmonization. The purpose of the method is to modify the DWI acquired at the target site, to correspond to the DWI of the same subject acquired at the reference site. In the training part, DWI from the target site is used as input and DWI from the reference site as ground truth, for patient *X*. Matched subjects are used to tune the weights of the harmonization network. During the forward phase, the network produces the predicted harmonized DWI that is compared with the corresponding expected DWI from the reference site. The difference between the predicted and the ground-truth (cost function) is back propagated into the network to update the weights in such a way that the loss decreases and the predicted harmonized DWI is closer to the ground truth. In the inference step, the trained network is used to generate the predicted harmonized DWI from unseen DWI data of the target site, which then become comparable to the DWI from the reference site.

The DL approach, which is illustrated in Figure 4, consists of two steps: (1) Training: the learning stage in which the network parameters are optimized using the DWI from the same subjects acquired in two sites (target and reference) and (2) Inference: the trained network is applied to harmonize all subjects of the target site.

The current deep learning algorithms for diffusion data harmonization are mainly based on spherical harmonic features. The aim is to bring all the images in the same SH domain, by modifying the SH coefficients of the target data creating harmonized DWIs of the target site that are comparable to the DWIs from reference site. To achieve this, the network is trained to generate the harmonized image starting from the image acquired at a target site, using the image acquired in the reference site as ground truth, as illustrated in Figure 4. Hence, diffusion data from subjects that were acquired in both reference and target sites are used for training the network. Once it is trained, the inference can be done for other subjects from the target site, to create harmonized images.

Tax et al. (2019) presented a summary of four deep learning algorithms and one sparse dictionary learning harmonization algorithm used to evaluate two harmonization tasks in diffusion MRI: scanner-to-scanner mapping and angular- and spatial-resolution enhancement, i.e., mapping between standard and state-of-the-art acquisitions. Each of the algorithms was built with different net architectures and strategies. The deep learning algorithms that were evaluated by Tax et al. (2019) are: spherical harmonic network (SHNet), spherical harmonic residual network (SHResNet), spherical network (SphericalNet), and fully convolutional shuffling network (FCSNet). The used SH coefficients, on which the net is based, are obtained starting from the diffusion signal of the same subjects scanned in different scanners and with different acquisition schemes. Here we summarize some of these methods. A more extensive benchmark can be found in Tax et al. (2019).

##### Spherical Harmonic Network (SHNet)

Spherical Harmonic Network is based on a classical Fully Connected Network (FCN) architecture, composed of a cascade of three fully connected layers, in which the rectified linear unity (ReLU) function is used as the activation function (Golkov et al., 2016; Koppers et al., 2017). Next, a batch normalization layer is used to stabilize. The different weights of the neural network layers are tuned by using paired images from different sites. The net is trained by matching data between the target site and the reference site to obtain the harmonized image. Once the network is trained, it can be used to harmonize unseen datasets from the target site. The main advantage of this network is that it is a simple FCN approach to tackle the harmonization problem. However, it might not be sufficiently sensitive to learn all the complex features of an accurate harmonization procedure.

##### Spherical Harmonic Residual Network (SHResNet)

A Convolutional Neural Network (CNN) approach has been presented by Koppers et al. (2018). In this case, the network algorithm is based on the novel concept of residual structure by He et al. (2016). This approach is based on the difference between the input and the ground truth (target signal). The main building blocks of SHResNet are so-called functional units consisting of three convolutional layers, where each functional unit predicts the coefficients of a single SH order (Koppers et al., 2017). The main advantage of using a residual network structure consists in the robustness against the degradation problem (decrease of accuracy due to the increased network depth) and hence enabling the use of a deeper network (more convolutional layers). Nonetheless, the harmonization is done per harmonic order of the SH signal, thus, the signal from both target and reference should have the same SH orders.

##### Spherical Network (SphericalNet)

SphericalNet is a novel deep learning approach based on spherical surface convolutions (Koppers and Merhof, 2018). It transforms the signal from SH space into spherical surface space, and performs three spherical surface convolutions. After each of these convolutions, a sigmoid activation function is applied in order to limit the signal’s range between 0 and 1 (Tax et al., 2019). The signal is converted back to SH space, followed by three 3-D convolutional layers with parametric ReLU as activation. Spatial information is combined in the last convolutional layer to project neighborhood info into one voxel. The advantage of this algorithm is that it uses spherical information during spatial convolution to improve accuracy in the harmonization procedure. However, for this algorithm the intensity signal has to be transformed twice (for SH domain and then to spherical surface domain), which could introduce additional complexity to the harmonization problem.

##### Fully Convolutional Shuffling Network (FCSNet)

Fully convolutional network is a patch-based deep learning harmonization algorithm inspired by Tanno et al. (2017). The architecture of this network contains four hidden convolutional layers with ReLU activation. Large patches are used as input, overlayed to cover the entire brain, and smaller patches are obtained as output. The last layer contains a “shuffle” operation and is composed of “skip” connections to increase the prediction accuracy. The cost function for this algorithm has two parts: channel-wise loss and loss on the function-value. The algorithm uses the patched-based fully convolutional network for diffusion data harmonization and resolution enhancement. One advantage of this approach is the use of large patches that inform about the local neighborhood and are beneficial for the harmonization procedure. On the other hand, neighborhood data could be biased and end up corrupting the harmonization algorithm.

Deep learning algorithms demonstrated the robust capability of solving non-linear problems such as data harmonization. However, some limitations are: (1) overfitting, i.e., when the model is more accurate in fitting known data but less accurate in predicting unseen data, (2) the need for a large amount of matched subjects scanned at different sites with similar acquisition sequences per site for training and (3) possible distortion of pathological information, if the net is trained with healthy subjects and then applied to patients.

### Method of Moments (MoM)

Method of Moments is a statistical harmonization approach that uses spherical moments to map DWI images from target to reference sites (Huynh et al., 2019). The first moment (*M*_1_) corresponds to the spherical mean and the second central moment (*C*_2_) corresponds to the spherical variance. The core idea is to match the spherical mean and spherical variance in order to correct for unwanted variability. Each voxel-wise n-th spherical moment (*M*_n_) is defined as the diffusion signal at constant *b*-value (*S*_b_) raised to the power of *n* integrated over all directions *g*: $M_{\text{n}}\left[S_{\text{b}}\right]=\int S_{\text{b}}^{\text{n}}\left(g\right)dg$. MoM matches *M*_1_and *C*_2_ per b-shell *b* using the mapping function (*f*_θ_): *M*_1_[*R*_b_] = *M*_1_[*f*_θ_(*T*_b_)] and *C*_2_[*R*_b_] = *C*_2_[*f*_θ_(*T*_b_)], where *R*_b_ is the diffusion signal acquired at the reference site, and *T*_b_ the signal at the target site. Considering the mapping function as *f*_θ = {α,β}_(*S*) = α*S* + β, α and β are the mapping coefficients calculated as $\alpha_{b}=\sqrt{\frac{C_{2}\left[R_{\text{b}}\right]}{C_{2}\left[T_{\text{b}}\right]}}$ and β_b_ = *M*_1_[*R*_b_]−α_b_*M*_1_[*T*_b_]. The MoM parameters are calculated in template space and then warped back to native space of the target subjects and applied to the DWI images. The MoM-harmonized DWI signal is $S_{\text{b}}^{\text{MoM}}=\alpha_{\text{b}}S_{\text{b}}+\beta_{\text{b}}$.

**FIGURE 5:**
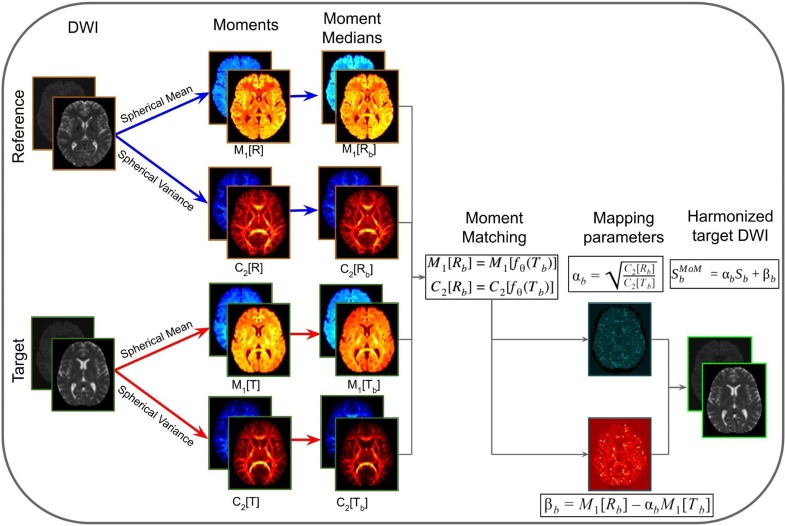
Representation of the method of moments harmonization pipeline. The purpose of the method is to modify the DWI of the target site, to correspond to the DWI acquired in the reference site. Initially, the diffusion signal in the reference (*R*) and target (*T*) are used to compute spherical means (*M*_1_[*R*] and *M*_1_[*T*]) and spherical variances (*C*_2_[*R*] and *C*_2_[*T*]) in native space for each b-shells (*b*). The spherical moments are warped to a common space, based on the target population. Then the moment medians are calculated across subjects (*M*_1_[*R*_b_], *C*_2_[*R*_b_], *M*_1_[*T*_b_], and *C*_2_[*T*_b_]). Afterward, the mapping parameters (α_b_ and β_b_) are calculated per b-shell, by matching the population moments. The mapping parameters are warped to native space and applied voxel-wise to the DWI images of target site subjects, obtaining the harmonized DWI.

The MoM approach is illustrated in Figure 5. In this method, *M*_1_ and *C*_2_ are computed in native space from the DWIs acquired in the reference and target sites. Next, the moment images are warped into a common space that is defined by the target data at the population level. Population moment median images across subjects are calculated for each of the moments for each of the sites. The mapping parameters (α and β) for the target site are obtained by matching the population median moments using the linear mapping function *f*_θ_. These parameters are warped to native space for each of the subjects of the target site and the mapping function is applied voxel-wise. Lastly, the harmonized DWI of the target data is obtained.

Advantages of the MoM are that it (1) allows direct harmonization of DWI images, without the need to represent them in any other space domain (e.g., SH space); (2) preserves directional information of the signal; (3) does not require that reference and target data have the same number of gradient directions; (4) does not require training data or matched populations with controls/patients, and (5) allows the harmonization of either a subject or a population of subjects. However, MoM as described in Huynh et al. (2019) does not harmonize multi-site data with different spatial resolution or different *b*-values. Possible solutions to cope with different spatial resolutions and different *b*-values would be to resample the reference data to the resolution of the target data, and rescale the signal, respectively, both prior to harmonization.

## Discussion

Multi-center and/or longitudinal studies using diffusion MRI data are significantly affected by inter- and intra-site variability. Sources of variability include, but are not limited to, hardware, acquisition settings, reconstruction algorithms, incompatible data formats and data quality. To cope with this variability, regulations and strategies are needed to facilitate harmonization of multi-center diffusion MRI data. In that respect, MR scanner vendors and researchers have a responsibility regarding the access and storage of DWI data, and transparency on reconstruction algorithms, acquisition protocols and applied pre- and post-processing steps. Ideally, worldwide governments should ally to enforce regulations regarding calibration procedures to MR scanner vendors. The use of the same quantitative calibration phantom and a standard procedure would decrease inter-scanner variability (Keenan et al., 2017; Prohl et al., 2019).

The need for harmonization has increased with the availability of large diffusion MRI multi-center datasets. Examples of these are the Human Connectome Project (HCP^2^), the Alzheimer’s Disease Neuroimaging Initiative (ADNI^3^), CENTER-TBI^4^, and the Cross-scanner and cross-protocol diffusion MRI data harmonization (Tax et al., 2019). For performing joint analysis of data that have been acquired with multiple acquisition settings, several statistical and mathematical harmonization approaches have been developed to reduce unwanted site variability while preserving the biological variability.

To overcome the challenges with respect to joint analysis of multi-center diffusion data, the scientific community has gathered to participate in challenges on data harmonization. The Diffusion MRI Data Harmonization^5^ 2017 and the Multi-shell Diffusion MRI Harmonization Challenge 2018 (MUSHAC^6^) were proposed with the aim to evaluate the performance of algorithms that enable the harmonization of DWI data. From the last challenge, Ning et al. (2019) presented a summary of results comparing the effects of DWIH methods on diffusion parametric maps. Different DWIH methods were used to harmonize the multi-shell DWI data. The algorithms range over three approaches: interpolation-based, regression-based and CNN algorithms. Diffusion parametric maps were calculated before and after the harmonization procedure, such as FA, MD, and MK. The results demonstrated that the harmonization algorithms are significantly effective in reducing the variability and maintaining the biological information.

**FIGURE 6:**
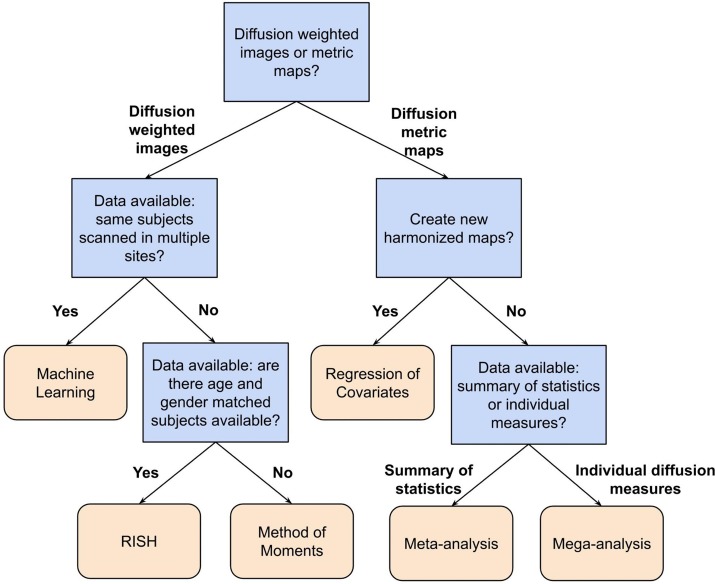
Flowchart describing a possible way to select a suitable harmonization method depending on the available data and research question at hand. In this flowchart, the first question to be answered is: Do you want to harmonize the DWI or the diffusion metric maps? For harmonization of the diffusion metric maps (right segment of the flowchart), the following question is: Do you want to create new harmonized metric maps? If so, the suggested harmonization approach would be one of the regression of covariates methods. In case of a negative answer, the next question is: Do you have individual measures available or a summary of statistics? If the user has a summary of statistics, the suggestion is to use a meta-analysis approach, otherwise, if one has individual diffusion measures, the suggestion is to harmonize the data using a mega-analysis approach. On the other hand, for harmonization of DWIs (left segment of the flowchart), the next question is: Do you have DWIs of the same subjects acquired in multiple sites? In case of an affirmative answer, the suggested approach is machine learning, which comprehends deep learning and sparse dictionary learning methods. In case of a negative answer, the following question is: Do you have DWIs of a cohort of subjects that is age- and gender-matched between the sites? If the user has matched data, the RISH method is suggested. Otherwise, the method of moments is the suggested approach.

In this paper, we have reviewed a variety of harmonization methods proposed in the literature. The decision as to which method to use depends on several aspects, such as the study design, the research question and the available data. In Table 1, we have categorized the reviewed methods in terms of their intrinsic properties. This categorization may help to select a harmonization method, given a certain diffusion MRI dataset and a specific research question. Additionally, Figure 6 shows a flowchart that could provide guidance for selecting the most appropriate harmonization strategy.

**TABLE 1:** Overview of the harmonization methods presented in this review.

Category	Method		References	Statistical harmoni- zation	Creates new harmoni- zed images	Individual measures required	Same subjects acquired in multiple centers required	Training data required	Similar acquisition protocols required	Inter subject co-registration of DWI required	Mapping parameters on template space
**DPMH**	Meta-analysis		Salimi-Khorshidi et al., 2009*;* Teipel et al., 2012*;* Jahanshad et al., 2013*;* Kochunov et al., 2014; Zhu et al., 2019	X							
	Mega-analysis			X		X					
	Regression of covariates	Human-phantom based harmonization (HuP)	Pohl et al., 2016	X	X	X			X		
		Hardware-phantom based harmonization (HaP)	Timmermans et al., 2019	X		X			X		
		Global Scaling (GS)	Fortin et al., 2017	X	X	X				X	
		Removal of Artificial Voxel Effect by Linear Regression (RAVEL)	Fortin et al., 2016	X	X	X				X	X
		Surrogate Variable Analysis (SVA)	Leek et al., 2012; Fortin et al., 2017	X	X	X				X	X
		Combined association test (ComBat)	Fortin et al., 2017	X	X	X				X	X
**DWIH**	Rotation Invariant Spherical Harmonics (RISH)		Mirzaalian et al., 2015, 2016, 2018; Karayumak et al., 2019		X	X		X	X	X	X
	Machine learning	Sparse Dictionary Learning (SDL)	St-Jean et al., 2016, 2017; Tax et al., 2019		X	X	X			X	
		Deep Learning (DL)	Golkov et al., 2016; Koppers et al., 2017, 2018; Tanno et al., 2017; Koppers and Merhof, 2018; Tax et al., 2019		X	X	X			X	
	Method of Moments (MoM)		Huynh et al., 2019	X	X	X				X	X

*Comparison between the methods related to: implementation of statistical harmonization, creation of new harmonized images, requirement of individual measures, requirement of images from the same subjects acquired in multiple centers, requirement of training data (i.e., matched subjects across sites are needed for obtaining the mapping between sites), requirement of similar acquisition protocols (i.e., diffusion directions, b-values, spatial resolution, TR, TE, SNR, etc.), requirement of inter subject co-registration, and implementation of mapping between sites through mapping parameters on template space. The X denotes if the method requires or performs the specific condition described in the column.*

For example, the flowchart can be applied to the study of Zavaliangos-Petropulu et al. (2019), who assessed the relation between diffusion MRI indices and cognitive impairment in brain aging using the ADNI3 dataset. In this study, new harmonized metrics maps (FA, MD, AD, and RD) were created using the ComBat method to remove any site-effects from the results. Following the flowchart presented in Figure 6, first, the research was related to the harmonization of diffusion metric maps, thus, the right segment of the chart is suggested to be followed. Next, the researchers aimed to create new harmonized maps, in this case the choice of a regression of covariates method was logical and appropriate. Along these lines, the suggested harmonization approach by our flowchart is in agreement with the decision from the authors.

In general, it is an ongoing challenge to define a gold standard for dMRI harmonization. A possible explanation for this might come from the complexity of removing the unwanted variability. The sources of unwanted variability may stem from differences in number of subjects acquired per site, MRI hardware, acquisition protocol (voxel size, repetition time, echo time, number of diffusion directions, number of b-shells, etc.), pre-processing steps and co-registration effects. In these circumstances, the preservation of expected biological variability is a useful criterion for evaluating the efficacy of harmonization methods, but this is only possible when the same subjects are scanned at different sites. When traveling human phantoms are included in the study design this provides a ground truth and allows for carefully evaluating the newly computed features and their accuracy and precision (Tax et al., 2019). However, traveling human phantoms datasets are mostly absent from a scenario of multi-center studies, where distinct subjects are scanned at different sites. Additionally, a note of caution in both cases is due here since anatomical differences or co-registration deformations (to a common space) may cause significant errors in the harmonization.

Although DPMH approaches have demonstrated their ability to harmonize diffusion metric measures for joint analysis in multi-center studies, there are some drawbacks, which can be avoided by using DWIH methods. First, DPMH methods require different transforms to harmonize each of the diffusion metrics of interest. This may have implications for multivariate analyses, as it is not guaranteed that subject-specific patterns (e.g., high FA in combination with low MD) are preserved after both metrics are harmonized separately. Second, DWIH methods do not rely on a specific diffusion model, hence unwanted variation is not propagated (and as a result made more complex) through model fitting. Moreover, any diffusion metric estimated from DWIH harmonized DWIs will automatically be harmonized as well. In this regard, DWIH approaches are more promising for reliable harmonization.

In a recent study by Cetin-Karayumak et al. (2019), DWIH was applied to harmonize diffusion MRI multi-site data prior to detection of white matter abnormalities in schizophrenia patients. RISH was retrospectively applied to DWIs of 13 different sites to remove the site-related differences. For this, a reference site was chosen and the DWI data from the other 12 sites were harmonized accordingly. The harmonization performance was evaluated in a group of matched controls, using their FA maps before and after harmonization. It was shown that the statistical differences between sites were removed and the inter-subject biological differences were preserved.

Nonetheless, many challenges remain for diffusion data harmonization in multi-center studies. Ideally, novel harmonization methods should not require training data of subjects scanned in multiple centers, and be applicable to data acquired with different spatial resolution, number of b shells, or number of diffusion gradient directions. Moreover, the availability of easily implementable methods and open-source platforms are important assets to encourage researchers to perform diffusion data harmonization in multi-center and longitudinal studies.

Furthermore, harmonization methods should be generalizable to clinical cases. Up to now, serious challenges that limit voxel-wise harmonization of DWI data of clinical patients are the co-registration requirement, since disease-related anatomical alterations may severely complicate co-registration, and the condition that the pathological content (e.g., diffusion properties of lesions) should be harmonized while the expected biological variability should not be affected. To overcome these limitations, the use of clinical data during the training of DWIH harmonization approaches would be valuable.

## Conclusion

While dMRI is routinely used in clinical workflows, comparing the signal intensity of dMRI scans across sites and over time is challenging. Harmonization methods aim to overcome this by recalibrating/recalculating either the DWI signal intensities or the resulting diffusion metrics. In this article an overview of harmonization methods in the literature was presented, covering meta- and mega-analysis, regression of covariates, rotation invariant spherical harmonics, machine learning algorithms and the method of moments. The proposed feature table and flowchart present the main characteristics of the methods, assisting in the decision of which method to use depending on the study design and the available data. Future developments of diffusion harmonization methods may benefit from focusing on DWIH approaches, avoiding unwanted variation propagates through diffusion model fitting.

## Author Contributions

MP and RP wrote the manuscript with comments from TB, PVD, P-JG, BJ, AR, AdD, and JS. MP and RP contributed equally to this manuscript. All authors read and approved the final manuscript.

## Conflict of Interest

The authors declare that the research was conducted in the absence of any commercial or financial relationships that could be construed as a potential conflict of interest.

## Footnotes

1 http://www.bic.mni.mcgill.ca/ServicesAtlases/ICBM152NLin2009

2 https://www.humanconnectome.org/

3 http://adni.loni.usc.edu/

4 https://www.center-tbi.eu/

5 https://projects.iq.harvard.edu/cdmri2017/challenge

6 https://projects.iq.harvard.edu/cdmri2018/challenge
